# Clinical and radiological characteristics of odontogenic orbital cellulitis

**DOI:** 10.1186/s12348-024-00422-0

**Published:** 2024-10-01

**Authors:** Vinay Tumuluri, Jessica Y. Tong, Krishna Tumuluri, Dinesh Selva

**Affiliations:** 1 Faculty of Health and Medical Sciences, School of Dentistry, University of Adelaide, Adelaide, Australia; 2 Department of Ophthalmology, Sydney Medical School, Faculty of Medicine and Health, Westmead Hospital, Save Sight Institute, The University of Sydney, Sydney, NSW Australia; 3 Department of Ophthalmology & Visual Sciences, University of Adelaide, North Terrace, 5000 Adelaide, South Australia Australia; 4 South Australian Institute of Ophthalmology, Royal Adelaide Hospital, Port Road, 5000 Adelaide, South Australia Australia

**Keywords:** Odontogenic orbital cellulitis, Orbital cellulitis, Odontogenic orbital abscess, Orbital abscess, Dental complications, Dental infections

## Abstract

**Purpose:**

To assess the radiological features and clinical outcomes of odontogenic orbital cellulitis.

**Method:**

Multi-centre retrospective study of odontogenic orbital cellulitis. Primary outcomes assessed were causal organism(s), clinical signs, radiological findings, management and visual outcomes.

**Results:**

Four patients with odontogenic orbital cellulitis were identified for inclusion. There was an equal proportion of men and women with a mean age of 43 years (range 25–56 years). All patients presented with an orbital compartment syndrome, with visual acuity of counting fingers (*n* = 1, 25%), hand movements (*n* = 1, 25%) and no perception of light (*n* = 2, 50%). The organisms implicated were *Streptococcus milleri* (*n* = 3, 75%) and *Streptococcus constellatus* (*n* = 1, 25%). MRI findings showed a subperiosteal abscess was present in all cases, which was characterised radiologically as a T1-hyperintense, T2 minimally hyperintense collection with restricted diffusion and a low apparent diffusion coefficient signal. Final visual acuity ranged from 6/6 to no light perception. One patient required an orbital exenteration due to extensive necrosis with sepsis and systemic deterioration.

**Conclusions:**

Odontogenic orbital cellulitis carries a serious risk of vision loss with a propensity to present with an orbital compartment syndrome secondary to *Streptococcus* species. Outcomes were highly variable, with two cases progressing to blindness of which one required an orbital exenteration.

**Supplementary Information:**

The online version contains supplementary material available at 10.1186/s12348-024-00422-0.

## Introduction

Odontogenic orbital cellulitis (OOC) represents 3–5% of orbital cellulitis cases [1], and tends to have a poor clinical course with complications including vision loss, septicaemia and cavernous sinus thrombosis [1–3]. The route for odontogenic spread of infection to the orbit can occur via the maxillary premolars and molars or spread via the infratemporal fossa up into the inferior orbital fissure. Oral pathogens are more likely to be associated with anaerobic organisms and the lack of empirical antibiotic coverage combined with its inherent virulence may result in more adverse outcomes. Therefore, prompt recognition of OOC is crucial to direct appropriate antimicrobial treatment and timing of surgical drainage. The existing literature is predominated by case reports highlighting the broad and unique microbiological profile of odontogenic organisms and associated vision loss [4–25]. This study presents a case series of 4 patients with OOC while examining the microbiological profile, clinico-radiological features and ophthalmic outcomes.

## Methods

The authors performed a retrospective study of patients admitted with OOC at major tertiary referral centres in Sydney and Adelaide, Australia. Study variables included data on demographics (age, sex, pre-existing medical conditions), ophthalmic findings (visual acuity, intraocular pressure, proptosis, dystopia, optic neuropathy), details of the odontogenic infection (dental procedure, intravenous/oral antibiotic use), investigations (fever, leukocytosis, inflammatory markers, blood cultures), radiographic findings (CT and MRI) and management. Institutional ethics approval was obtained and the study followed the tenets of Declaration of Helsinki.

## Results

The clinical summary of each case is presented below and further summarised in Tables 1 and 2. A literature review of all published reports of odontogenic orbital cellulitis is provided in Supplementary Table 1.

**Table 1:** Clinical characteristics of OOC

	Case 1	Case 2	Case 3	Case 4
**Age/Gender**	25/M	38/M	53/F	56/F
**Dental History**	None	Exodontia of upper molar (16)	Broken UL molar (27, 28)	Exodontia of 26
**Blood Cultures**	Nil growth	*Streptococcus constellatus*	Nil growth	*Streptococcus milleri*
**Initial**,** Final Visual Acuity**	NPL → NPL	HM → 6/7.5	NPL → N/A	CF → 6/6
**Causal Organism(s) wound swab**	*S. Milleri 2+*	*Streptococcus constellatus*	*S. Milleri 1+*,* mixed anaerobic flora 2+*	*Streptotoccus milleri*
**Surgical Management**	Lateral canthotomy/cantholysis Exodontia of 47, drainage of dental abscess, drainage of orbital abscess via upper lid skin crease approach and lower lid transconjunctival approach	Lateral canthotomy/cantholysis Orbital abscess drainage and functional endoscopic sinus surgery	Lateral canthotomy/cantholysis Exodontia of 27, 28, orbital abscess drainage via functional endoscopic sinus surgery Further orbital abscess drainage and exploration + external frontoethmoidectomy Lid sparing orbital exenteration	Lateral canthotomy/cantholysis Orbital subperiosteal abscess drainage + Lynch incision + Caldwell-Luc approach to drain ethmoidal and maxillary sinuses Further drainage of orbital abscess + functional endoscopic sinus surgery Further drainage of sinuses and temporal abscess via Gilles incision
**Number of Procedures during acute admission**	1	1	3	3
**Delayed Procedures**	Nil	Nil	Repair of nasoorbital medial fistula with a temporalis muscle flap	Lateral canthoplasty and ectropion repair

CF = count fingers; F = female; HM = hand movements; M = male; NPL = no perception of light

**Table 2:** Radiological characteristics of OOC.

	Case 1	Case 2	Case 3	Case 4
**CT features on admission**				
**Location of subperiosteal abscess**	Orbital floor, posterior lateral wall and medial wall	Orbital floor and medial wall	Orbital floor and roof	Orbital floor, posterior lateral wall and superolateral orbit
**Extraocular muscle enlargement**	Inferior Rectus	Inferior Rectus and Lateral Rectus	Inferior rectus	Lateral Rectus and Superior Rectus
**Retrobulbar fat stranding**	Present	Present	Present	Present
**Orbital emphysema**	Yes	Nil	Nil	Yes
**MRI features**				
**T1 weighted intensity**	No MRI	Isointense	Isointense	Hypointense
**T2 weighted intensity**		Hyperintense signal along orbital floor and adjacent to the inferior rectus	Hyperintensity and enlargement of inferior rectus muscle	Hyperintensity of medial and lateral orbital regions
**Contrast enhancement**		Moderate	None	Moderate

### Case presentations

#### Case 1

**Fig. 1:**
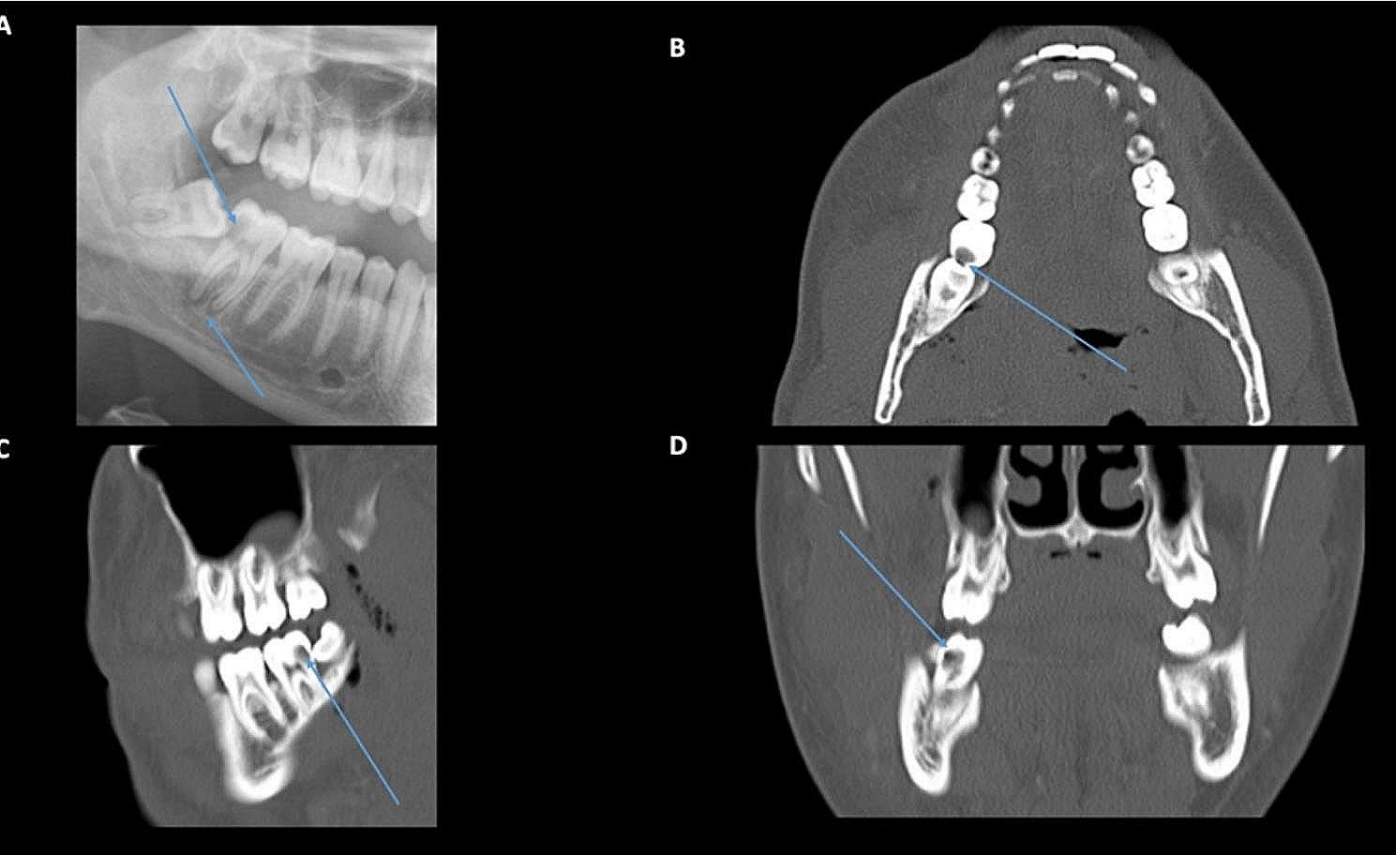
Case 1: OPG and CT facial bone scans. **A**: OPG image showing extensive carious damage on the crown of the lower right molar (47) and periapical radiolucency suggesting infectious spread through alveolar bone (arrows). **B-D**: There is extensive radiolucency on 47 distal

**Fig. 2:**
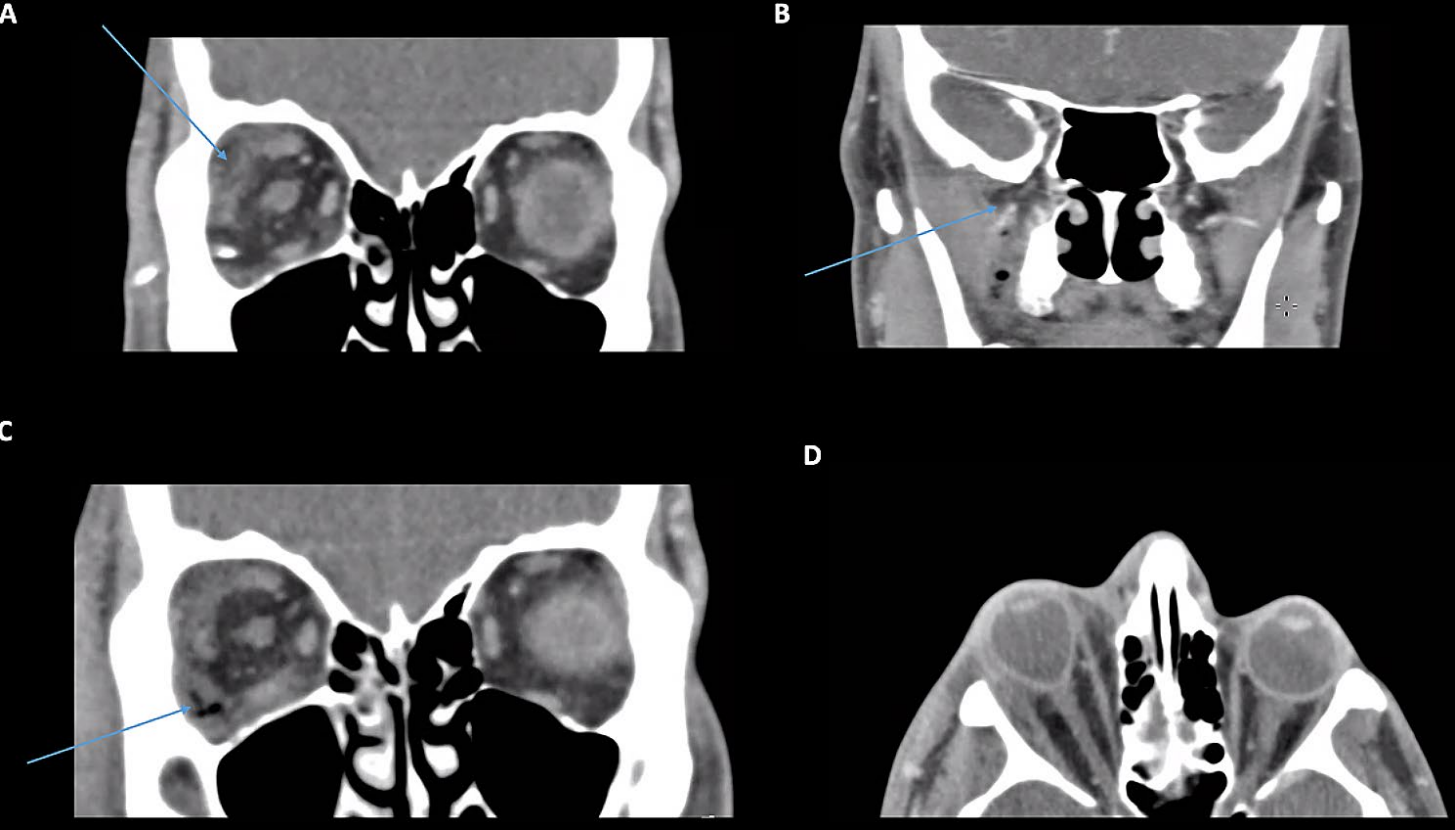
Case 1: CT orbital scans. **A**: Coronal image highlighting extensive subperiosteal abscess involving the right superolateral and inferolateral regions of the orbit. **B**: Soft tissue enhancement with alteration of the fat signal within the pterygopalatine and infratemporal fossae. **C-D**: A repeat CT orbital scan 2 days later demonstrating the presence of orbital emphysema with proptosis and tenting of the right globe

A 25-year-old Caucasian male was admitted with a 3 day history of right tooth and jaw pain with progressive periorbital oedema. On examination, visual acuity of the right eye was no perception of light (NPL) with raised intraocular pressure (IOP) of 50mmHg, 6 mm of proptosis and a relative afferent pupillary defect (RAPD). Orthopantomogram (OPG) showed an abscess of the second lower right molar (47) with periapical lucency and widening of the periodontal ligament (PDL) space (Figs. 1 and 2). CT orbital scans demonstrated an extensive subperiosteal abscess of the right orbital floor, lateral wall, and roof with soft tissue enhancement of the infratemporal fossa and parapharyngeal space. A lateral canthotomy and cantholysis was performed. The patient underwent urgent drainage of the subperiosteal abscess in conjunction with extraction of the right molar tooth (47) and drainage of the dental abscess. Intraoperative orbital swabs showed growth of *Streptococcus milleri* sensitive to penicillin. The patient was treated with a combination of IV ceftriaxone, vancomycin, tazocin and meropenem. He was discharged 12 days post-admission with no improvement in vision. At 5 months follow-up, the right eye vision remained no light perception with evidence of optic atrophy.

#### Case 2

**Fig. 3:**
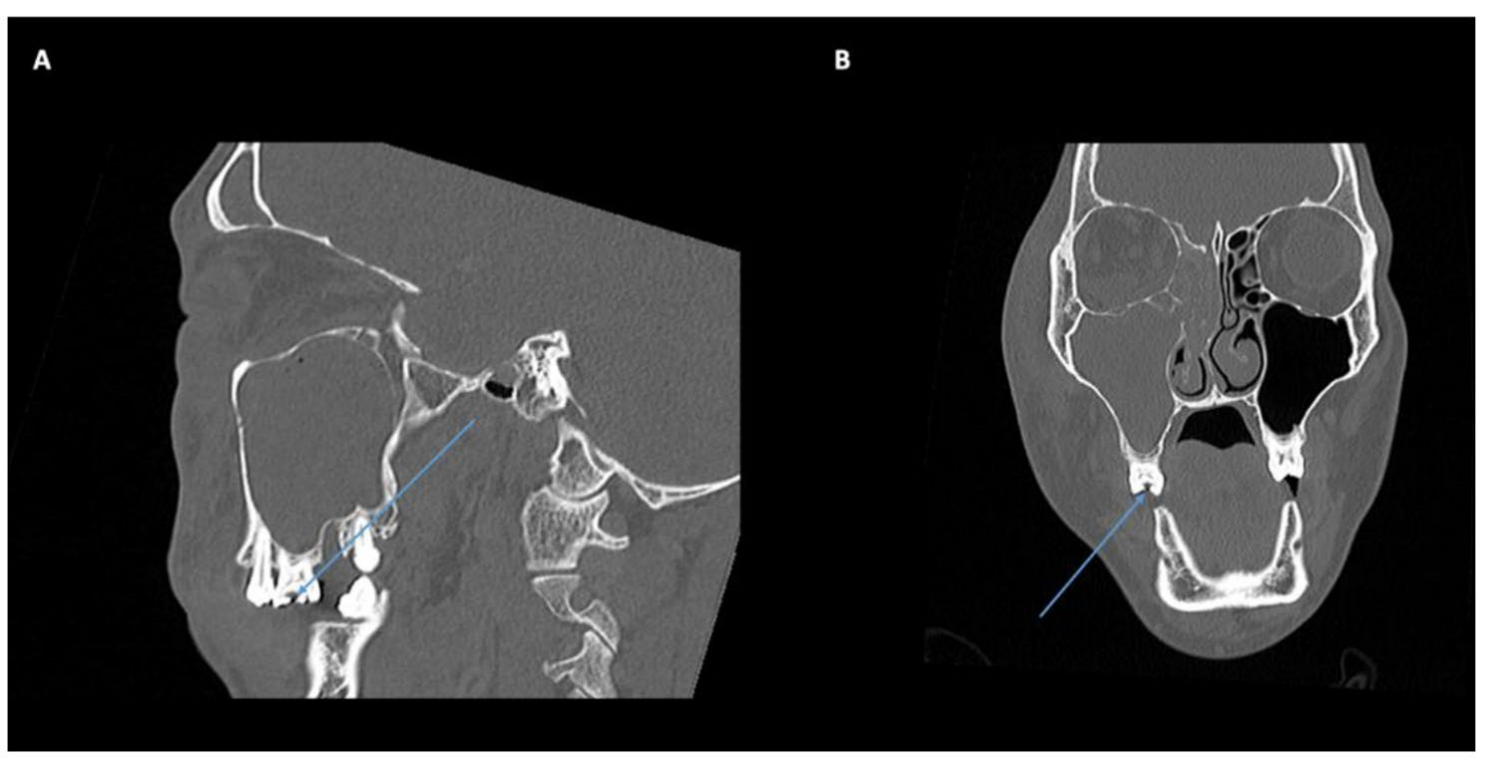
Case 2: The odontogenic source of infection is demonstrated on the CT facial bones imaging. **A-B**: sagittal and coronal CT images demonstrating hypoattenuation of the upper right molar (16) occlusal, and secondary opacification of right maxillary sinus

**Fig. 4:**
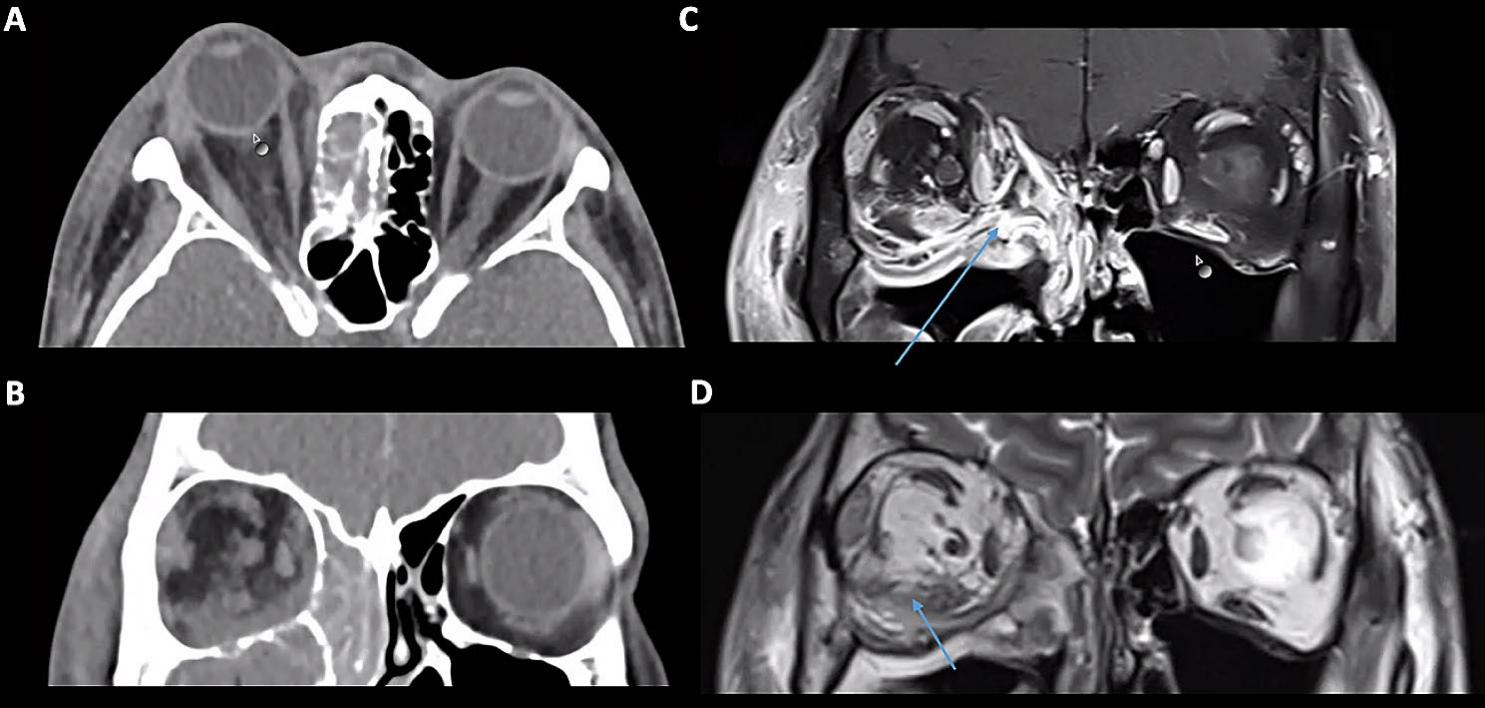
**A-B**: CT orbital scans. There is significant right sided proptosis with retrobulbar fat stranding and a subperiosteal abscess along the medial wall and orbital floor. Opacification of the right ethmoid and maxillary sinuses are also demonstrated. **C**: MRI orbital scan performed several days following admission. T1 fat-suppressed contrast-enhanced MRI demonstrating an enlarged and enhancing right inferior rectus and avid enhancement in the region of the subperiosteal abscess. **D**: Coronal T2 image shows hyperintensity of the right inferior rectus and adjacent subperiosteal abscess collection

A 38-year-old Chinese man presented with a 1 day history of right eye pain, periocular swelling and erythema. The patient had extraction of an upper right molar tooth (16) 1 day prior and presented with almost complete ophthalmoplegia. On presentation, his visual acuity was hand movements with an IOP of 45 mmHg. Orbital compartment syndrome necessitated an emergent lateral canthotomy. Under the guidance of Infectious Diseases, he was commenced on IV amoxicillin/clavulanic acid and metronidazole on admission and within 24 h was changed to vancomycin and piperacillin/tazobactam, given the possibility of polymicrobial infection. Orbital CT scans revealed enhancement of the inferior and lateral rectus muscles and presence of a right orbital floor and medial wall subperiosteal abscess. There was also occlusal lucency with widening of periodontal ligament of 16 and opacification of the right maxillary sinus (Figs. 3 and 4). He underwent a functional endoscopic sinus surgery and subperiosteal abscess drainage. Blood cultures revealed *Streptococcus constellatus*,* Citrobacter koseri*,* Streptococcus anginosus* and mixed anaerobes. Progress MRI scans were performed and revealed persistent inflammation along the orbital floor, adjacent to the inferior rectus. There were no intracranial complications. At final follow up 13 months later, his vision had recovered to 6/7.5 with preservation of optic nerve function.

#### Case 3

**Fig. 5:**
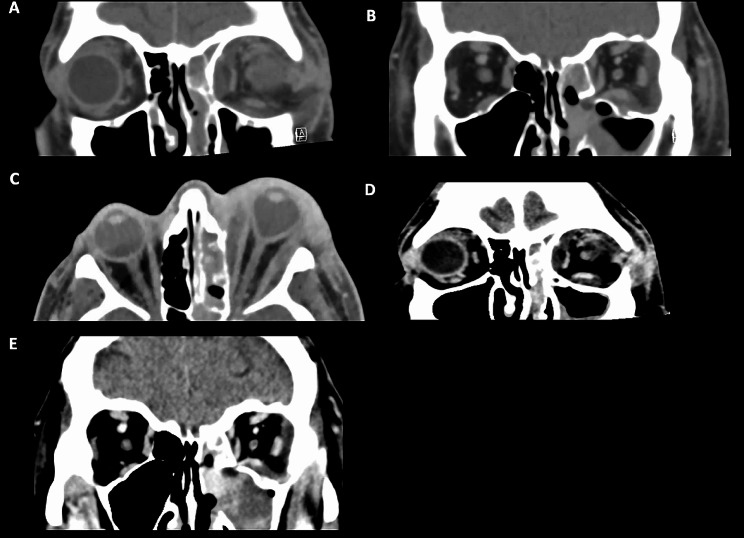
Case 3: **A-B**: Coronal CT image demonstrating subperiosteal collection along the left medial wall, orbital floor and lateral wall. **C**: Axial CT image demonstrating significant tenting of the left globe. **D-E**: A repeat CT orbital scan revealed persistent circumferential subperiosteal collections of the left orbit

A 53-year-old Caucasian female presented with a 1 day history of vomiting, left periorbital swelling and vision loss. She had a broken left upper tooth 4 days prior to admission. On examination, visual acuity was no light perception in the left eye, IOP 60mmHg with an RAPD. CT orbital imaging demonstrated a left orbital floor and medial subperiosteal abscess (Fig. 5A-C). The patient was commenced on IV ceftriaxone and metronidazole and underwent left orbital subperiosteal abscess drainage with endoscopic sinus surgery. Intraoperative swabs confirmed *Streptococcus milleri* with mixed anaerobic flora. The patient had persistent septicaemia and repeat CT imaging confirmed persistent subperiosteal abscesses involving all four walls of the left orbit (Fig. 5D-E). Further drainage was performed and intraoperatively, there was extensive abscess formation and necrosis of the orbital tissue, which was debrided. Dental extraction of the upper left molar teeth (27, 28) was simultaneously performed. Despite this, the patient remained haemodynamically unstable with continued septicaemia and requiring inotropic support and intubation in ICU. Following multidisciplinary discussion regarding the extensive necrosis of orbital tissue and poor response to antibiotics despite 2 weeks of intensive treatment, the patient underwent a left orbital exenteration and external fronto-ethmoidectomy. Her systemic condition improved immediately. Within 24 h of exenteration, she was taken off inotropic support and extubated, and was discharged 10 days later. One year post exenteration she underwent a temporalis muscle flap and closure of a naso-orbital fistula.

#### Case 4

**Fig. 6:**
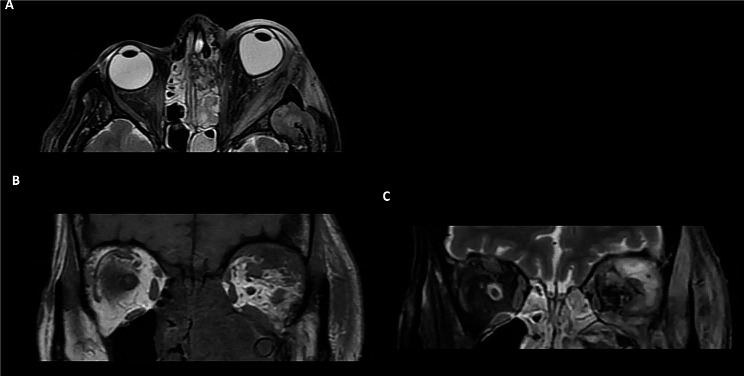
Case 4: **A**: MRI image demonstrating left sided proptosis with tenting of the globe. **B-C**: There is an abscess centred around the left superolateral and inferior orbit that is T1 isointense and T2 hyperintense, with associated enlargement and oedema of the lateral and inferior recti

A 56-year-old Caucasian female was admitted to a peripheral hospital for management of left orbital cellulitis 2 weeks following dental extraction of the left upper molar tooth (26). At presentation, she had visual acuity of count fingers in the left eye, IOP 35mmHg with 8 mm of proptosis and an RAPD. CT orbital imaging showed mild enlargement of the left lateral and superior recti with retrobulbar fat stranding (Fig. 6). She underwent an immediate lateral canthotomy and cantholysis and was admitted for commencement of IV Cephazolin and Metronidazole. The maxillofacial team performed drainage of the left ethmoid and maxillary sinuses via a Lynch incision and Caldwell Luc approach, and a swinging lid approach was performed for drainage of the orbital floor and lateral subperiosteal abscess. Wound swabs confirmed *Streptococcus Milleri*. The patient was slow to respond and had persistent periorbital oedema, high intraocular pressures and sepsis. She underwent repeat orbital abscess drainage and transferred to a tertiary centre. MRI orbital scans confirmed left superior and lateral orbital abscesses that demonstrated T1 hypointensity with a T2 hyperintense signal. She underwent further drainage of a residual abscess involving the ethmoid and maxillary sinuses and temporal fossa. The patient self-discharged 10 days after admission with visual acuity of count fingers. She was continued on oral Moxifloxacin and at 6 months follow-up, visual acuity had improved to 6/6.

## Discussion

Odontogenic orbital cellulitis (OOC) represents only 3–5% of all orbital cellulitis cases but is generally associated with a poor prognosis pertaining to worse visual outcomes and increased risk of serious complications such as vision loss and cavernous sinus thrombosis [1]. This series demonstrates that patients with OOC more frequently present with an acute orbital compartment syndrome with vision loss and optic neuropathy, and the organism most commonly implicated is *Streptococcus milleri*. While the patients in this series did not experience any intracranial complications, repeated surgical interventions were necessary and visual prognosis was generally poor in cases secondary to *Streptococcus milleri*.

The spread of odontogenic infection to the orbit occurs via several pathways [1]. The most common mode of spread occurs via the maxillary sinus. The buccal plate is a thin layer of bone separating the maxillary premolars and molars from the maxillary sinus and dehiscence can facilitate rapid spread into the orbit via the orbital floor. Odontogenic orbital cellulitis is typically preceded by a periapical infection of carious maxillary molars and premolars [26]. Root proximity of these dentition to the maxillary sinus floor mucosa, serves as a conduit for the passage of bacterial organisms. Spread can also occur via the retromaxillary soft tissue and extend into the infratemporal fossa, inferior orbital fissure and finally into the orbit. In particular, the proximity of this portion of the inferior orbital fissure to the orbital apex can place patients at greater risk of visual impairment. The spread of odontogenic orbital cellulitis to the surgical orbital apex, defined as the posterior 3/5 of the retrobulbar orbital space, makes for a vision-threatening condition [27]. Infectious spread to this region presents higher risk of optic nerve decompression due to the most significant decrease in volume, attesting to the important of early recognition of clinical and radiological signs. Thrombophlebitic extension to the valveless pterygoid venous plexus can result in septic emboli and cavernous sinus thrombosis.

### Clinical characteristics

Odontogenic orbital cellulitis can cause rapid vision loss with a poor prognosis for recovery. Youssef et al. reported 24 combined cases of odontogenic orbital cellulitis from the literature in 2008, of which 11 (45.8%) of which recorded a final vision of light perception/no light perception [18]. Similarly, of the 4 cases presented in our series, 1 (25%) recorded a final visual acuity of no light perception and one patient underwent an exenteration.

The thin nature of the buccal plate which sits atop the maxillary alveolar bone promotes the rapid spread of odontogenic infections via the maxillary sinus [1]. There is great variability in time to presentation with OOC – Bullock et al., from reported ranges from 48 h to 13 days [1]. In our series, the duration from dental infection to orbital cellulitis ranged from 24 h to 2 weeks. The delayed presentation may be due to partial treatment with oral antibiotics soon after a dental extraction.

### Microbiological profile and virulence factors

The microbiological profile of OOC consists of a mix of anaerobic and aerobic bacteria [28]. In Umeshappa et al.’s series of 100 patients with infection of the odontogenic space, *Staphylococcus aureus*,* Streptococcus viridans and Streptococcus milleri* were amongst the most common causative anaerobic bacteria [29]. The trends over time have remained consistent, with a review of the current literature revealing that 68% of cases are due to gram-positive and non-motile *Streptoccoccus* bacteria [1, 4–18, 20–22, 24, 30–39]. Importantly, much of these infections are polymicrobial and a combination of anaerobic and aerobic bacteria comprise 60% of odontogenic infectious flora [40] (Supplementary Table 1).

Despite residing as commensal flora within the oral cavity, the *Streptococcus milleri* species is virulent and a prominent cause of abscess formation [41]. Virulence factors specific to *S. Milleri* include adherence, invasion, spreading factors, cell wall proteins and component histadine kinases [42]. Unlike other mucosal streptococcal species, *S. milleri*, is more frequently associated with men [41]. In fact, male gender has been positively correlated with vision loss [18].

### Radiological characteristics

A hallmark radiological finding of dental infection is the presence of lucency surrounding the root apex as well as a widening of the periodontal ligament (PDL). The presence of a dental subperiosteal abscess can appear radiologically similar to periapical lucency [2]. Widening of the PDL space occurs due to the presence or spread of periodontal pathogens [43]. Additionally, carious tooth damage, which serves as an inlet of bacteria, presents as a hypoattenuation within the crown.

Inflammation of the maxillary sinus can create a conduit for the spread of infection through the midface. Orbital inflammatory signs are apparent in the context of cellulitis, including extraocular muscle enlargement and retrobulbar fat stranding [44], which was present in all patients within our series. Orbital emphysema is another radiological sign of anaerobic infection due to a path of communication between the infected maxillary sinus and orbit, allowing the spread of anaerobic bacteria such as the streptococcal species. When considering all causes of orbital cellulitis, the medial rectus is the most commonly affected muscle likely due to adjacent involvement of the ethmoid sinuses [45] (Supplementary Table 1). In contrast, half of the patients in our series presented with lateral rectus enlargement, which would be more consistent with an inferolateral pathway of pathogenic spread from the maxillary soft tissue, infratemporal fossa and inferior orbital fissure in OOC.

Most *Streptococcus* species are facultative anaerobes and therefore can present with evidence of gas within the orbit on CT scans. Gas may be an indication of sinogenic spread of infection into the orbit, or it may be due to gas-producing organisms. Two cases (50%) in this study demonstrated orbital emphysema secondary to *Streptococci milleri.* In addition to anaerobic *Streptococcus*, other organisms implicated include *Clostridium*,* Proteus*,* Klebsiella* species and *Escherichia coli* [33, 46, 47]. Gas within the orbit has significant potential for vision loss via raised intraorbital pressure leading to tissue ischaemia and optic neuropathy [47]. Therefore, orbital emphysema is an ominous radiographic sign that should warrant concern for an aggressive organism.

Magnetic resonance imaging (MRI) is superior for its delineation and monitoring of soft tissue changes and abscess formation in the brain and orbit. However, in the acute setting, access can be limited and thus CT imaging predominated as the initial imaging of choice in our series. All patients in this study eventually had an MRI scan, which showed T2 hyperintensity in the regions of involvement, which is consistent with oedema and inflammation.

## Management

### Surgical Management

There are various factors that predispose patients to urgent surgical intervention. These include compromised vision, elevated IOP, proptosis greater than 5 mm, unilateral maxillary sinus opacification suspicious for dental aetiology [4, 28]. Emergent canthotomy and cantholysis as well as surgical drainage was performed in all 4 of our patients. Exodontia (removal of the tooth from the underlying alveolar bone) is the optimal approach as apicectomy can lead to recurrent bacterial spread [48]. Drainage of an orbital subperiosteal abscess, and endoscopic sinus surgery is often performed concurrently to address maxillary and any additional paranasal sinusitis [49]. External approaches may also be performed for sinus drainage. In this series, the Caldwell-Luc approach and a medial Lynch incision was used to approach the maxillary and ethmoidal sinus respectively in 1 case. Furthermore, 1 patient required an orbital exenteration due to extensive necrosis of the orbital soft tissues.

### Visual prognosis

The average time between initial presentation to final follow-up was 18 months (range: 5–30 months) in this series. Two of the 4 patients in this study had a final visual acuity of 6/6 or 6/7.5, while the remaining half of the cohort had poor outcomes of NPL vision or requirement for an orbital exenteration. A review of the literature demonstrated that 34.5% of all patients had a final vision of LP or NPL (Supplementary Table 1). [1, 2, 5, 18, 25, 38, 39, 50]

## Conclusions

Odontogenic orbital cellulitis is a serious condition with significant potential for sight and life-threatening complications. Periapical radiolucency and widening of the periodontal ligament are concerning radiological features that suggest the presence of a dental infection. Orbital emphysema, retrobulbar fat stranding and a hyperintense T2 signal were other common radiological features. *Streptococcus milleri* and polymicrobial isolates are often implicated in OOC. Prompt surgical management and close surveillance for ocular, intracranial and systemic complications via a multidisciplinary approach is of the utmost importance.

## Electronic supplementary material

Below is the link to the electronic supplementary material.

Supplementary Material 1

## Data Availability

No datasets were generated or analysed during the current study.

## Abbreviations

OOC: Odontogenic orbital cellulitis
NPL: No perception of light
IOP: Intraocular pressure
RAPD: Rapid afferent pupillary defect
OPG: Orthopantomogram
PDL: Periodontal ligament
CT: Computerised tomography
MRI: Magnetic resonance imaging
