# Supplementary material for: Clinical and radiological characteristics of odontogenic orbital cellulitis
Source: J Ophthalmic Inflamm Infect. 2024 Oct 1;14:48. doi: 10.1186/s12348-024-00422-0 (PMC11445216; doi:10.1186/s12348-024-00422-0)
Supplement: Supplementary file 1 — Supplementary Material 1 [file 12348_2024_422_MOESM1_ESM.docx]

**Supplementary Table 1.** Literature Review.

| Author | Age | Sex | Initial Vision | Final Vision | Oral/IV Antibiotics | Sinus Involvement | Orbital Involvement | Orbital Microorganisms | CT findings | MRI findings | Time between  dental infection  to OOC | Surgical Management |
| --- | --- | --- | --- | --- | --- | --- | --- | --- | --- | --- | --- | --- |
| Allegrini D. et al.  [4] | 46 yo | F | 20/20 | NS | IV piperacillin/tazobactam | Nil | Ophthalmoplegia, proptosis | Streptococcus constellatus | Cavernous sinus thrombosis | Distended cavernous sinus and superior ophthalmic vein with lack of enhancement | 6 hours | Nil |
| Sheils, C et al.  [5] | 65 yo | M | 20/400, NLP | 20/150, NLP | NS | Nil | Bilateral multifocal orbital abscesses | Polymicrobial organisms consistent with oral flora | Multifocal orbitofacial abscess  Midface abscess of the upper alveolar origin  Proptosis and tenting of the globe | No MRI | 21 days | Bilateral orbit and facial abscess drainage |
| White, M et al.  [6] | 25 day old | M | NS | NS | NS | Maxillary and ethmoidal sinusitis | Subperiosteal abscess of orbital floor and medial wall with proptosis | Staphylococcus aureus | Maxillary and ethmoid sinus opacification with displaced right maxillary molar tooth root | High T2 signal  with enhancement along inferior and medial extraconal space. | NS | Drainage of neonatal tooth and subperiosteal abscess |
| Rothschild, M et al.  [7] | 49 yo | M | 20/20 | 20/20 | IV aztreonam (due to penicillin allergy), vancomycin, and trimethoprim-sulfamethoxazole | Nil | Inferior orbital fissure abscess with severe 8mm proptosis | Streptococcus anginosus | Left side odontogenic abscess with hypodensities indicative of gas in ipsilateral masseter, temporalis, pterygopalatine fossa at the inferior orbital fissure | No MRI | NS | Canthotomy, cantholysis  Orbital and temporalis muscle abscess drainage |
| Eltayeb, A et al.  [8] | 35 yo | M | NS | NS | IV ceftazidime and metronidazole | Nil | Subperiosteal abscess of orbital roof with proptosis | NS | Soft tissue swelling and gas in the subcutaneous area of left buccal region extending to zygomatic region up to orbital roof | No MRI | 5 days | Buccal abscess drainage |
| Stead, T et al.  [9] | 26 yo | M | 20/25 RE, | NS | IV clindamycin | Maxillary, frontal and ethmoidal sinusitis | Right proptosis with medial subperiosteal abscess | Microaerophilic streptococci | Right proptosis with complete opacification of the right maxillary, ethmoid and frontal sinuses. | No MRI | 6 days | Tooth extraction and functional endoscopic sinus surgery |
| Li, E et al.  [10] | 69 yo | F | CF RE | CF RE | Oral fluconazole    IV vancomycin, ciprofloxacin, and metronidazole | Nil | Right proptosis with orbital abscesses | Streptococcus constellatus | Dental and orbital abscesses | Abscesses were noted to be centrally non-enhancing. Restricted diffusion within the posterior optic nerve was indicative of an optic neuropathy. |  | Debridement of extensive necrotic orbital and periorbital tissues |
| Mansour, A et al.  [11] | 59 yo | M | 6/7.5 | NS | Oral amoxicillin clavulanate | Maxillary sinusitis | Soft tissue swelling around the left lacrimal sac | Enterococcus | Complete opacification of maxillary sinus with soft tissue swelling extending to the anterior orbit inferiorly | Non-enhancement of multiple loculated collections in the left mandible up to the maxillary region  Lucency present around the root of the 28 tooth | 24 hours | Drainage of tooth abscess and medial canthus incision |
| Vu, Q et al.  [12] | 67 yo | M | 20/200 | 20/25 +2 | Methylprednisolone 125mg with IV metronidazole, ceftriaxone, and vancomycin | Maxillary sinusitis | Enlargement of inferior rectus | NS | No CT | Diffuse enhancement of the left intraorbital optic nerve and sheath with mild stranding of fat  Enlargement of inferior rectus | 24 hours | Molar tooth extraction |
| Yan, W et al.  [31] | 26 yo | M | 6/6 | 6/6 | IV flucloxacillin, ceftriaxone, and metronidazole | Maxillary and ethmoidal sinusitis | Right inferomedial orbital abscess with 6.5mm of proptosis | Staphylococcus Epidermidis | Right inferomedial orbital abscess with gas and soft tissue stranding  Periapical lucency around roots of 16 with periodontal abscess | No MRI | 2 days | Inferior transconjunctival orbitotomy for abscess drainage and functional endoscopic sinus surgery |
| Arunkumar, K et al.  [13] | 10 yo | M | NS | NS | IV van-comycin, meropenem and metronidazole | Maxillary sinusitis | Extraconal subperiosteal abscesses of superomedial and inferolateral orbit | NS | Complete opacification of maxillary sinusitis with hypodense collections within orbit indicative of gas, enlargement of inferior rectus | No MRI | NS | Carious tooth extraction, pus evacuation and sinus lavage, stab incision to drain infra-orbital pus |
| Procacci, P et al.  [14] | 35 yo | M | NS | NS | NS | Maxillary, ethmoidal and frontal sinusitis | Left sided proptosis with intraorbital gas collection | No isolates | Opacification of the ethmoid and maxillary sinus with erosion of the left orbital floor.  Enlargement of extraocular muscles and intraorbital gas | Gas and high T2 signal of tissue within the superior and superomedial extraconal space | NS | Tooth extraction, left maxillary sinus drainage via Caldwell-Luc approach and orbital abscess drainage |
| Tavakoli, M et al.  [15] | 30 yo | M | CF | 20/20 | IV ceftriaxone, vancomycin, and metronidazole | No sinus involvement | Left intraconal abscess with compression of the globe | NS | Discrete intraconal abscess with heterogenous density and marked impression on the globe with proptosis | No MRI | 3 days | Extraction of infected tooth, extroral incison to drain dental abscess, subcilliary incision to drain orbital abscess |
| Park, C et al.  [16] | 41 yo | M | 20/100 RE, 20/20 LE | No change | IV ceftriaxone, clindamycin, methylprednisolone | Maxillary sinusitis | Right proptosis with tenting of the globe | Staphylococcus epidermidis | Severe sinusitis in the right maxilla and inflammation of the right temporalis and mastication muscles Right severe proptosis with fat stranding of intraconal space and lateral rectus enlargement | No MRI | 4 days | Maxillary sinus drainage |
| De Medeiros, E et al.  [17] | NS | F | NS | NS | Amoxicillin 875 mg and clavulanic acid 125 mg | Sphenoid and ethmoidal sinusitis | NS | NS | Extensive carious decay of tooth #26 | No MRI | 7 days | Maxillary sinus drainage via the Caldwell-Luc approach, |
| Youssef, O et al.  [18] | 62 yo | M | NLP | NLP | Amphotericin B, vancomycin, and piperacillin/tazobactam | Pansinusitis | Right proptosis with optic neuropathy | B-hemolytic Streptococcus group F | Right pansinusitis with hypodensity suggestive of air within the intraconal space, severe proptosis with tenting of globe | No MRI | 4 days | Canthotomy, cantholysis  Right endoscopic maxillary antrostomy, anterior and posterior ethmoidectomy |
| Youssef, O et al. | 22 yo | F | 20/25 | 20/20 | IV clindamycin | Left frontal and ethmoid sinusitis | Large superior subperiosteal abscess with retromaxillary soft tissue swelling extending into the inferior orbital fissure | No isolate | No CT | Large superior subperiosteal abscess, hyperintense on T2, | 4 days | Sub-brow incision to drain subperiosteal abscess |
| Youssef, O et al. | 18 yo | M | NLP | NLP | IV vancomycin and ampicillin/sulbactam | Left maxillary and ethmoidal sinusitis | Intraorbital abscess | Alpha- and beta-hemolytic Streptococcus, Staphylococcus (coagulase negative), and Prevotella buccae (Bacteroides) | Left maxillary and ethmoid sinus opacification, orbital emphysema with reticulation of fat and tenting of globe | No MRI | 3 days | Canthotomy and cantholysis  Left maxillary antrostomy, total ethmoidectomy  Molar extraction, orbitotomy for debridement of necrotic fat |
| Thakar, M et al.  [20] | 34 yo | F | 6/6 | 6/6 | IV ciprofloxacin and metronidazole | Nil | Abscess in the posterior-inferior orbit | NS | Diffuse, mildly enhancing soft tissue mass in posterior-inferior orbit engulfing the inferior rectus and extending close to the optic nerve | No MRI | 3 days | NS |
| Allan, B et al.  [21] | 20 yo | M | 20/30 | 20/20 | IV penicillin G and metronidazole | Right ethmoid and maxillary sinusitis | Inferior extraconal abscess with enlargement of the right inferior and medial rectus muscles | Fastidious streptococci & staphylococcus (coagulase negative) | Right ethmoid and maxillary sinus opacification with indistinct bone margins suggesting periostitis with dehiscence of right orbital floor. | No MRI | 5 days | Maxillary sinus antrostomy |
| Goswami P et al.  [22] | 50 yo | M | 6/4 | NS | IV ertapenem, moxifloxacin and oral amoxicillin/clavulanic acid | Nil | Right sided proptosis | Campylobacter rectus | Intraorbital and extraconal heterogeneous soft tissue lesion involving the lacrimal gland with osteolytic involvement of the greater wing of the sphenoid | Superotemporal extraconal orbital and temporal fossa abscess, associated localised dural enhancement. | NS | Anterior orbitotomy revealing purulent fluid of the superolateral orbit |
| Grimes, D et al.  [23] | 31 yo | F | 6/12 | NS | IV cefuroxime and metronidazole | Left maxillary and ethmoid sinusitis | Left sided proptosis | NS | Complete opacification of the left maxillary and ethmoid sinuses | No MRI | Within 30 days | NS |
| Blumenthal, D et al.  [24] | 3 yo | M | NS | NS | IV penicillin G and ampicillin | Right maxillary and ethmoid sinusitis | Orbital swelling | Group B streptococcus | Soft tissue densities in the retro-orbital space and sinuses | No MRI | NS | Right ethmoidectomy, middle turbinate resection, ethmoidectomy and maxillary sinus drainage |
| Arat et al.  [25] | 55 yo | F | CF both eyes | NLP | IV vancomycin, ceftriaxone, and clindamycin |  | Cavernous sinus thrombosis with bilateral proptosis | Fusobacterium nucleatum | Cavernous sinus enlargement with bilateral proptosis | Abnormal enhancement and enlargement of the cavernous sinuses bilaterally with narrowing of the cavernous segments of the internal carotid arteries  Filling defect in right superior ophthalmic vein likely representing intraluminal thrombus | NS | Bifrontal craniotomy and decompression of the left optic nerve within the bony canal |
| Ogundiya et al. [32] | 35 yo | F | 20/40 | 20/30 | IV penicillin, clindamycin, nafcillin, cefotaxime, | Left maxillary sinusitis | Lateral subperiosteal abscess enveloping lateral rectus muscle | Alpha haemolytic *Streptococcus* and mixed anaerobes | Enlargement of left rectus muscles, abscess in pterygomandibular space with development of globe tenting and proptosis | No MRI | 11 days | Drainage of pterygomandibular space, exodontia of 27 |
| Bullock et al. ^[1]^ | 19 yo | F | 20/200 | 20/20 | Oral penicillin, dicloxacillin, IV ampicillin | Pansinusitis | Superomedial subperiosteal abscess | Beta haemolytic *Streptococcus* | NS | No MRI | 5 days | Drainage of subperiosteal abscess |
| Bullock et al. ^[1]^ | 35 yo | M | NS, pupil reflex initially intact | NLP | IV methicillin, gentamicin, clindamycin | Left maxillary sinusitis | NS | *Enterobacter aerogenes, micrococci, pneumococci* | NS | No MRI | 13 days | Extraction of 28, orbital and maxillary sinus abscess drainage |
| Flood et al. ^[33]^ | 30 yo | M | 20/20 | 20/20 | Oral penicillin, IV penicillin, IV gentamicin | Left maxillary sinusitis | Inferolateral subperiosteal abscess | *Bacteroides melaninogenicus* | Displacement of the lateral rectus by subperiosteal abscess.  Intraconal abscess with gas and significant left proptosis | No MRI | 7 days | Incision and drainage of abscess within left buccal space  Exodontia of 24, 25, 26, 27, 28  Left Caldwell Luc approach and nasal antrostomy to drain left infratemporal space  External approach to drain pterygopalatine space  Drainage of inferolateral subperiosteal abscess |
| Henry et al. ^[34]^ | 27 yo | F | 20/20 | 20/20 | IV Ceftriaxone, metronidazole, imipenum/cilastatin, metronidazole | Right maxillary, frontal and ethmoid sinusitis | Subperiosteal abscess along orbital roof and medial wall | *Bacteroides loeschii, Peptostreptococcus* | Opacification of right maxillary, ethmoidal and frontal sinuses. Gas in the orbit lateral to lamina papyracea extending to the roof. | No MRI | 2 days | Exodontia of 16, external and endonasal drainage of ethmoidal and maxillary sinuses, drainage of right orbital abscess |
| Janakarajah et al. ^[35]^ | 14 yo | M | 20/20 | 20/20 | IV ampicillin and metronidazole | Right maxillary sinusitis | NS | *Staphylococcus albus* | NS | No MRI | 4 days | Exodontia of 16, drainage of maxillary sinus |
| Bizakis et al. ^[36]^ | 40 yo | F | 20/25 | 20/20 | Oral ampicillin | Right ethmoid sinusitis | Inferior and close to apex of orbit | *Streptococcus Milleri* | Retrobulbar abscess with air-fluid level, opacification of the right ethmoid sinus | No MRI | 2 days | Caldwell Luc approach for drainage of the maxillary sinus |
| Pavlovici et al. ^[37]^ | 55 yo | M | CF | 20/70 | Vancomycin, ceftriaxone, metronidazole, anticoagul | Normal sinus involvement | Superior ophthalmic vein thrombosis | *Streptococcus anginosus* | Distension and central filling defect of right superior ophthalmic vein, bilateral prominence of cavernous sinus, enlargement of extraocular muscles and intraconal fat stranding with thrombosis | No MRI | 4 days | NS |
| Caruso et al. ^[2]^ | 5 patients in case series, individual details not specified |  |  |  | NA | NA | NA | NA | Periapical lucency (5/5), subperiosteal abscess (2/5), mild to severe premolar soft tissue swelling (5/5) with sinus opacification (5/5) | No MRI | NA | NA |
| Moschos et al. ^[38]^ | 38 yo | M | NLP | NLP | Amoxicillin and clavulanic acid, amikacin, clindamycin, benzylpenicillin | Left maxillary, ethmoid sinusitis | Proptosis | *Streptococcus constellatus* | Opacification of left maxillary and ethmoidal sinus with erosion of the medial antral wall | Enhancement of the retrobulbar fa^t^ with proptosis | 3 days | Caldwell Luc approach for antral drainage, lateral orbital incision to decompress the orbit and drain retrobulbar space |
| Patt et al. ^[39]^ | 25 yo | M | 20/50 | LP | IV penicillin, tobramycin, chloramphenicol | Left maxillary, ethmoid sinusitis | NS | *Staphylococcus aureus* | Left maxillary and ethmoid sinuses, stranding of retrobulbar fat | No MRI | 14 days | Exodontia of left maxillary molar, Caldwell Luc approach to drain maxillary/ethmoid sinus |
| Sharma et al. ^[51]^ | 60 yo | F | NS | NS (described as ‘intact vision’) | IV cefotaxime and metronidazole, tobramycin, IV piperacillin-tazobactam | Temporal and infratemporal fossa abscess formation | Proptosis | NS | NS | T2 hyperintensity of retrobulbar intraconal fat with blurred margins around the optic nerve sheath and proptosis | NS | Lateral and medial canthotomy to decompress orbit, drainage of temporal/infratemporal space, exodontia of carious molars in right maxilla |
| Sharma et al. ^[51]^ | 41 yo | F | NS | NS (described as ‘intact vision’) | Oral amoxicillin-clavulanic acid and metronidazole | NS | NS | NS | NS | No MRI | 2 days | Exodontia of 24 tooth (upper left first premolar) |

CF = counting fingers; F = female; M = male; NA = not applicable; NLP = no light perception; NS = not specified; yo = years old
